# β-Adrenoceptor activation depresses brain inflammation and is neuroprotective in lipopolysaccharide-induced sensitization to oxygen-glucose deprivation in organotypic hippocampal slices

**DOI:** 10.1186/1742-2094-7-94

**Published:** 2010-12-20

**Authors:** Tina Markus, Stefan R Hansson, Tobias Cronberg, Corrado Cilio, Tadeusz Wieloch, David Ley

**Affiliations:** 1 Pediatrics, Lund University, Lund, Sweden; 2 Obstetrics & Gynecology, Lund University, Lund, Sweden; 3 Neurology, Lund University, Lund, Sweden; 4 Cellular Autoimmunity, Malmo University Hospital, Malmo, Sweden; 5 Laboratory for Experimental Brain Research, Lund University, Lund, Sweden

## Abstract

**Background:**

Inflammation acting in synergy with brain ischemia aggravates perinatal ischemic brain damage. The sensitizing effect of pro-inflammatory exposure prior to hypoxia is dependent on signaling by TNF-α through TNF receptor (TNFR) 1. Adrenoceptor (AR) activation is known to modulate the immune response and synaptic transmission. The possible protective effect of $\tilde{\alpha }$ and $\tilde{\beta }$AR activation against neuronal damage caused by tissue ischemia and inflammation, acting in concert, was evaluated in murine hippocampal organotypic slices treated with lipopolysaccharide (LPS) and subsequently subjected to oxygen-glucose deprivation (OGD).

**Method:**

Hippocampal slices from mice were obtained at P6, and were grown *in vitro *for 9 days on nitrocellulose membranes. Slices were treated with β1(dobutamine)-, β2(terbutaline)-, α1(phenylephrine)- and α2(clonidine)-AR agonists (5 and 50 μM, respectively) during LPS (1 μg/mL, 24 h) -exposure followed by exposure to OGD (15 min) in a hypoxic chamber. Cell death in the slice CA1 region was assessed by propidium iodide staining of dead cells.

**Results:**

Exposure to LPS + OGD caused extensive cell death from 4 up to 48 h after reoxygenation. Co-incubation with β1-agonist (50 μM) during LPS exposure before OGD conferred complete protection from cell death (P < 0.001) whereas the β2-agonist (50 μM) was partially protective (p < 0.01). Phenylephrine was weakly protective while no protection was attained by clonidine. Exposure to both β1- and β2-agonist during LPS exposure decreased the levels of secreted TNF-α, IL-6 and monocyte chemoattractant protein-1 and prevented microglia activation in the slices. Dobutamine remained neuroprotective in slices exposed to pure OGD as well as in TNFR1^-/- ^and TNFR2^-/- ^slices exposed to LPS followed by OGD.

**Conclusions:**

Our data demonstrate that activation of both β1- and β2-receptors is neuroprotective and may offer mechanistic insights valuable for development of neuro-protective strategies in neonates.

## Background

Perinatal hypoxia-ischemia remains an important cause of brain damage which may result in long-term impairment including cerebral palsy and mental retardation. Hypoxia-ischemia occurs as result of disturbed gaseous exchange between mother and fetus, most commonly occurring during labor at birth.

The normal transition from fetal to neonatal life, *ie *birth, is associated with a surge of circulating catecholamines (CA) which is several-fold higher than during normal conditions [1]. We have shown that preterm infants exhibit a circulating anti-inflammatory response with a homogenous increase in IL-10 during the first postnatal hours [2]. We speculated that CA release at very preterm birth may be causal in generating a circulating anti-inflammatory response. This is supported by *in vitro *studies showing that exposure of both peripheral immune cells and microglia to adrenergic agonists causes a suppression of stimulated release of the pro-inflammatory cytokine TNF-α with an increase in IL-10 [3,4].

Fetal hypoxia *per se *is associated with an increase in circulating CA. The main source of circulating CA is the fetal adrenomedullary system [5]. The chromaffine cells of the adrenal medulla are directly sensitive to low pO_2_, elevated pCO_2 _and decreased pH resulting in release of norepinephrine (NE) and epinephrine. Of note, the adrenomedullary response to these stressors, which accompany fetal asphyxia, is functional during the perinatal period and is decreased in later development following splanchnic innervation of the adrenal medulla. The surge of NE and E has been shown to be essential for cardiovascular compensation to hypoxia with centralization of blood flow to vital organs. Inability to increase adrenal perfusion during induced fetal hypoxia is associated with cardio-vascular collapse and fetal death [6].

The capacity to generate a stress response during hypoxia/ischemia may be of vital importance beyond that essential for hemodynamic adaptation. Increased levels of CA in the CNS may serve an important role in endogenous protection against inflammation and ischemia. From locus coeruleus and nuclei in the brain stem, the cerebral cortex including the hippocampus, is widely innervated by noradrenergic fibers. Several findings support the hypothesis that monoamines provide neuroprotection against ischemia by acting at cell populations implicated in the development of ischemic neuronal damage. In microglia, as in peripheral immune populations, NE induces immune-suppression by c-AMP dependent mechanisms, characterised by reduced cytokine release [7,8]. Norepinephrine also directly modulates the excitability in neuronal cells and regulates the release of neurotransmitters in the hippocampus [9-12]. Furthermore, monoamines promote neuronal survival by inducing release of neurotrophins in astrocytes [13].

Inflammation and ischemia have a synergistic damaging effect in the immature brain [14] and animal studies show that induced inflammation prior to ischemia, depending on the applied time interval between the respective insults, may heavily aggravate resulting neuronal damage [15,16].

We have recently developed a model of induced inflammation and "ischemia" in the neonatal brain using juvenile murine organotypic hippocampal slice cultures. We have shown that exposure to lipopolysaccharide (LPS) immediately prior to oxygen-glucose deprivation (OGD) heavily aggravated cell death in the neuronal subregions, CA1, CA3 and the dentate gyrus (DG) [17]. The sensitizing effect of LPS to OGD was dependent on signalling by tumor necrosis factor (TNF) -α through TNF receptor 1 (TNFR1). Absence of TNFR1 was associated with decreased levels of secreted TNF-α during LPS exposure. Thus, signalling by TNF-α is a key mechanism in inflammatory induced sensitization to ischemia. Of note, neuronal cell death following OGD with LPS pre-exposure could be completely blocked by a NMDA-receptor antagonist showing that signalling by TNF-α through TNFR1 resulted in enhanced excitotoxic cell death [17].

Adrenoceptor (AR) stimulation has both immuno-modulating effects and interacts with excitoxic mechanisms in cell populations within the brain [3,9]. We therefore hypothesized that AR stimulation may provide neuroprotection in LPS-induced sensitization to OGD in the immature hippocampus. The organotypic hippocampal slice culture offers the study of CNS parenchyma in an intact three-dimensional tissue architecture and the evaluation of the integrated response of various cellular elements [18,19]. This is an advantage as both ischemic damage and protection provided by immuno-modulation is thought to result from activation of multiple cell types in the brain and their interactions. In addition, the effect of AR stimulation can be studied locally within brain tissue excluding effects from cardiovascular changes.

We investigated the effect of α1-, α2-, β1- and β2-AR activation respectively on neuronal cell death following OGD with LPS pre-exposure in the immature murine hippocampus. To characterise neuronal damage we measured levels of cell death over time. Immune response was characterised by determining levels of released cytokines and by histological evaluation of microglial morphology. To evaluate if neuroprotection by AR activation depended on modified signalling through TNFRs we performed separate studies in slices from mice devoid of TNFR1 and TNFR2 respectively.

## Methods

### Organotypic hippocampal tissue cultures

All animal experiments were approved by the Malmo/Lund ethical committee on animal experiments (approval number M 73-04). Offspring from date-mated balb/c mice (Harlan, Scandinavia, Denmark) and C57BL/6 TNFR1^-/- ^and TNFR2^-/- ^mice were used for experiments. In house breeding generated TNFR1^-/- ^and TNFR2^-/- ^mice as described previously [17]. In brief, hippocampal organotypic tissue cultures were prepared essentially according to the method of Stoppini, as described earlier [18,20], from 6-day-old mice. In short, hippocampi were dissected in ice-cold Hank's balanced salt solution, with 20 mmol/L HEPES (4-(2-hydroxyethyl)-1-piperazineethanesulfonic acid), 100U penicillin-streptomycin per milliliter, and 3 mg/mL D-glucose and cut into 250-μm-thick slices using a McIllwain Tissue Chopper. Sections were plated onto Millicell culture inserts, one per insert (0.4 mm Millicell-CM, 12 mm in diameter, Millipore Corp, Bedford, MA, USA). Slices were cultured in 35°C and 90% to 95% humidity in a CO_2 _incubator for 9 days before experiment. The culture medium consisted of 50% modified Eagle's medium with Earle's balanced salt solution (MEM), 25% horse serum, and a 18% Hank's balanced salt solution and was supplemented with 4 mmol/L L-glutamine, 50 units penicillin-streptomycin per milliliter, and 20 mmol/L D-glucose. The pH was adjusted to 7.2 using NaHCO_3_. During the first week of culture, 2% of the supplement B27 was included in the medium and thereafter omitted and replaced with the same volume of water. On *in vitro *day 9, when the experiment was initiated, horse serum was omitted and the volume was replaced with MEM. This medium was used throughout the experimental protocol. Before experimental start, the culture medium was changed the day after the preparation and thereafter three times a week. All substances were obtained from Invitrogen (Carlsbad, CA, USA) with the exception of D-glucose, which was from Sigma-Aldrich (St Louis, MO, USA).

### Experimental protocol

All cultures used in one experiment were prepared from mice pups from one to two females with litters born on the same day. Experiments were started on DIV9, and slices were assorted into four groups with one slice from each individual mouse per experimental group and six slices per group. Slices were incubated with propidium iodide (PI, Sigma-Aldrich) for 1 h to assess the background levels of cell death. Photomicrographs were taken using fluorescent light microscopy and slices were again sorted to include only undamaged slices displaying no PI uptake. Light microscopy was used to include only slices with an organotypic anatomy. Propidium iodide was present in the medium throughout the experimental protocol and all experiments were carried out in serum-free medium. Experimental groups that were investigated are as follows: Control = cultures exposed to medium changes at corresponding time points as experimental groups, preincubation period of 24 h followed by transfer to fresh medium for 48 h; LPS +OGD = incubation with LPS (1 μg/mL; Sigma-Aldrich) for 24 h followed by oxygen-glucose deprivation (OGD) and thereafter transfer to fresh medium for 48 h; Oxygen-glucose deprivation (OGD) = preincubation without LPS for 24 h, followed by OGD and thereafter transfer to fresh medium for 48 h.

The effect of adrenergic agonists on neuronal cell death was evaluated following LPS+OGD. The preferential β1-agonist dobutamine (5, 10 or 50 μM, Sigma, Saint Louis, MO, USA), the preferential β2-agonist terbutaline (5, 10 or 50 μM, Sigma), the α1-agonist phenylephrine (5 or 50 μM, Sigma) and the α2-agonist clonidine (5 or 50 μM, Sigma) were added in separate experiments during the pre-incubation period of 24 h with LPS.

**Figure 1:**
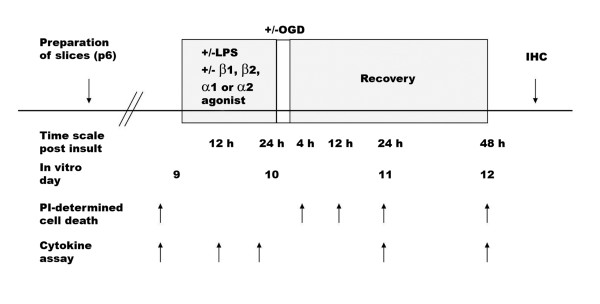
**Schematic illustration of experimental design**. LPS = lipopolysaccharide; PI = propidium iodide; OGD = oxygen-glucose deprivation; IHC = immunohistochemistry; β1-agonist = dobutamine; β2-agonist = terbutaline; α1- agonist = phenylephrine; α2-agonist = clonidine.

In further experiments, β1- and β2-receptor specific effects were evaluated by co-incubating the preferential β1-agonist dobutamine with either CGP 20712A (50 μM, Sigma), a selective β1-antagonist, or ICI 118551 (50 μM, Sigma), a selective β2-antagonist, during the pre-incubation period as described above. An overview of the experimental protocol is given in Figure 1.

The effect of β1-agonist dobutamine on neuronal cell death following pure OGD (excluding LPS exposure prior to OGD) was evaluated separately with the aim of evaluating effects on primarily excitotoxic cell death. Further, the effect of dobutamine on cell death following LPS + OGD was evaluated in slices from TNFR1^-/- ^and TNFR2^-/- ^mice in order to assess the role of signaling by TNF-α through TNFR1 and TNFR2, respectively.

Before OGD, cultures were washed once in pre-warmed phosphate-buffered saline (PBS) and transferred to the anaerobic incubator (Elektrotek Ltd, Keighley, UK) in an empty pre-warmed plate according to previously described protocols for OGD [20]. The anaerobic incubator had an atmosphere comprising 10% H_2_, 5% CO_2_, and 85% N_2_, and the temperature was maintained at 35 ± 0.3°C. Inside the incubator, the slices were transferred to wells containing pre-equilibrated OGD medium. The OGD medium consisted of, in mmol/L concentrations, 2 CaCl_2_, 125 NaCl, 25 NaHCO_3_, 2.5 KCl, 1.25 NaH_2_PO_4_, 2 MgSO_4_, and 10 sucrose and had a pH of 7.4. After 15 mins of OGD, the slices were transferred to fresh oxygenated culture medium and placed in the CO_2 _incubator. In all groups, slices were cultured for 48 h after the time point of OGD, and photomicrographs were taken in parallel at 4, 12, 24, and 48 h after OGD.

### Quantification of cell damage

Propidium iodide was used as a marker for cell death and was included in the medium in all groups throughout the experimental protocol [20]. Photomicrographs of PI-stained slices were obtained before experimental start and at time points of 4, 12, 24, and 48 h after OGD in all groups. Densitometric analysis was performed on photomicrographs to obtain quantitative values of cell death. Propidium iodide intensity was measured using a standardized area of interest applied over the CA1 region. The CA1 region provided the most consistent and reproducible damage following the insults. Background uptake was measured in an undamaged area outside the CA2 region in each individual slice, and values of cell death in regions of interest were obtained by subtracting the levels of mean fluorescent intensity in the background region in each slice from the mean fluorescent intensity values in the region of interest. For image processing, the commercial software Image-Pro Plus 4.0 (Media Cybernetics, MD, USA) was used.

### Immunohistochemistry

Slices were fixed for immunohistochemistry at 48 h after OGD. Unsectioned slices were immunostained for the microglial marker F4/80. Slices were fixed with 4% paraformaldehyde in PBS for 10 mins and then stored at 4°C in PBS until used. Endogenous peroxidase activity was quenched with 3% hydrogen peroxide in 10% methanol for 30 mins. After rinsing, slices were blocked with normal swine serum and diluted in PBS with 1% Triton X-100 for 1 h before overnight incubation with a primary antibody, monoclonal rat anti-F4/80 (Serotec, Kidlington, Oxford, UK, diluted 1:1,000 in PBS containing 5% normal rabbit serum and 1% Triton X-100). After rinsing, slices were incubated with a biotinylated secondary antibody, rabbit anti-rat IgG antibody. The secondary antibody was diluted at 1:1,000 in PBS containing 1% Triton X-100 and swine serum for 90 mins. After rinsing, slices were incubated with the avidin-biotin-peroxidase complex (Vectastain Elite, Vector Laboratories Inc., Burlingame, CA, USA) for 1 h before reaction with the chromogen 3,3 diaminobenzidine supplemented with 0.06% nickel (DAB-Safe; 0.5 mg/mL; Saveen-Werner AB, Malmo, Sweden). Serum and the secondary antibody and the avidin-biotin-peroxidase complex (Vectastain Elite) were from Vector Laboratories. Micrographs of immunostained slices were prepared using a microscope (Olympus BX51) equipped for brightfield microscopy with a digital camera (Olympus U-PMTVC). Figures show original, unmodified photomicrographs.

### Cytokine measurements

Levels of secreted cytokines in culture medium were determined at 12 and 24 h after the start of LPS pre-incubation with or without dobutamine/terbutaline and at 12, 24, and 48 h after OGD. Samples of medium (50 μL) were immediately frozen on dry ice and were thereafter stored at -80°C until analyzed in one batch. Levels of TNF-α, monocyte chemoattractant protein 1 (MCP-1) and interleukin (IL)-6 were determined by cytometric bead assay (Becton Dickinson (BD) Biosciences, San Jose, CA, USA) and flow cytometry according to the manufacturer's recommendations and as described earlier [2]. Two-color flow cytometric analyses were performed using a FACSCalibur Flow Cytometer (BD Biosciences). Data were acquired and analyzed using BD Biosciences CBA software. The lower limit of detection for the various cytokines evaluated ranged from 2 to 10 pg/mL. For results above the upper limit of detection, serial dilution of the sample was performed to accurately determine cytokine levels. A level of 0.1 pg/mL was regarded as non-detectable.

### Statistical analysis

Statistical analysis was performed using SPSS v14.0 for Windows. Data are expressed as mean ± SEM. All compared groups were run in parallel inside the anaerobic incubator. Differences between groups were assessed using ANOVA for repeated measures with *post hoc *Bonferroni correction for multiple comparisons. Variability between experimental dates was adjusted for by including date as a variable. *P*-values of <0.05 were considered significant.

## Results

### Neuronal cell death following lipopolysaccharide exposure and oxygen-glucose deprivation

**Figure 2:**
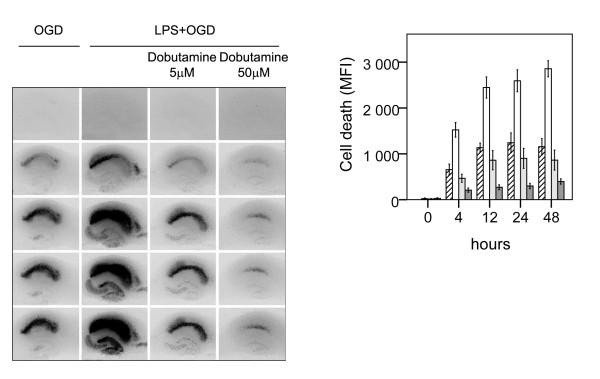
**Effect of β1-agonist dobutamine on neuronal cell death following lipopolysaccharide-induced sensitization to OGD in murine hippocampal slice cultures**. A. Temporal development of cell damage (mean fluorescence intensity (MFI)) after 15 min oxygen-glucose deprivation (OGD), LPS (1 μg/ml) for 24 h + 15 min oxygen-glucose deprivation (OGD), β1-agonist dobutamine, (5 μM) + 24 h LPS + 15 min OGD and β1-agonist dobutamine (50 μM) + 24 h LPS + OGD. The same culture is shown at repeated time points of recovery. B. Cell death (MFI) after 15 min OGD (hatched bars), 24 h LPS+ 15 min OGD (white bars), dobutamine (5 μM) + 24 h LPS + 15 min OGD (shaded bars) and dobutamine (50 μM) + 24 h LPS + 15 min OGD (dark shaded bars) at repeated points of recovery. Data are shown as means ± SEM with n = 18. LPS+OGD *versus *OGD, P < 0.001 in the CA1; dobutamine (5 μM) + LPS + OGD *versus *LPS + OGD, p < 0.01 in the CA1; dobutamine (50 μM) + LPS + OGD *versus *dobutamine (5 μM) + LPS + OGD, p < 0.001 in the CA1. Repeated-measures ANOVA with *post hoc *Bonferroni.

Representative photomicrographs and mean values of cell death illustrating aggravated neuronal cell death following LPS + OGD as compared to pure OGD are shown in Figure 2.

Cell damage induced by OGD only, was seen from 4 h, and reached its maximal level by 12 h of reoxygenation, (hatched bars). Pre-incubation with LPS during 24 h before OGD severely aggravated cell damage as compared to sole exposure to OGD (p < 0.001 respectively) (white bars). The time course of cell damage was similar to pure OGD. Sole exposure to LPS did not damage cells in the CA1 region up to 72 h following exposure. Control slices exhibited no cell damage.

### Protective effect of adrenergic agonists on neuronal death following lipopolysaccharide exposure and oxygen-glucose deprivation

**Figure 3:**
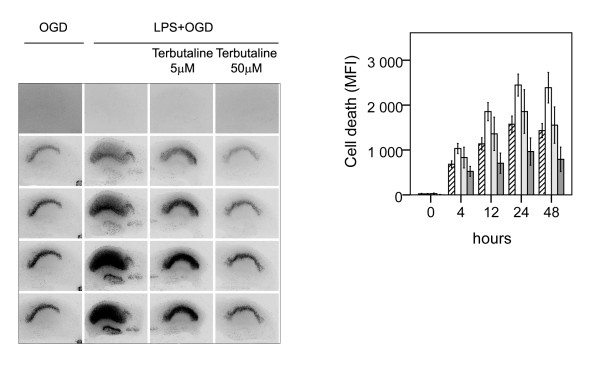
**Effect of β2-agonist terbutaline on neuronal cell death following lipopolysaccharide-induced sensitization to OGD in murine hippocampal slice cultures**. A. Temporal development of cell damage (mean fluorescence intensity (MFI)) after 15 min oxygen-glucose deprivation (OGD), LPS (1 μg/ml) for 24 h + 15 min oxygen-glucose deprivation (OGD), β2-agonist terbutaline, (5 μM) + 24 h LPS + 15 min OGD and β2-agonist terbutaline (50 μM) + 24 h LPS + OGD. The same culture is shown at repeated time points of recovery. B. Cell death (MFI) after 15 min OGD (hatched bars), 24 h LPS+ 15 min OGD (white bars), terbutaline (5 μM) + 24 h LPS + 15 min OGD (shaded bars) and terbutaline (50 μM) + 24 h LPS + 15 min OGD (dark shaded bars) at repeated points of recovery. Data are shown as means ± SEM with n = 18. LPS+OGD *versus *OGD, P < 0.001 in the CA1; terbutaline (5 μM) + LPS + OGD *versus *LPS + OGD, p < 0.01 in the CA1; terbutaline (50 μM) + LPS + OGD *versus *terbutaline (5 μM) + LPS + OGD, p < 0.01 in the CA1. Repeated-measures ANOVA with *post hoc *Bonferroni.

**Figure 4:**
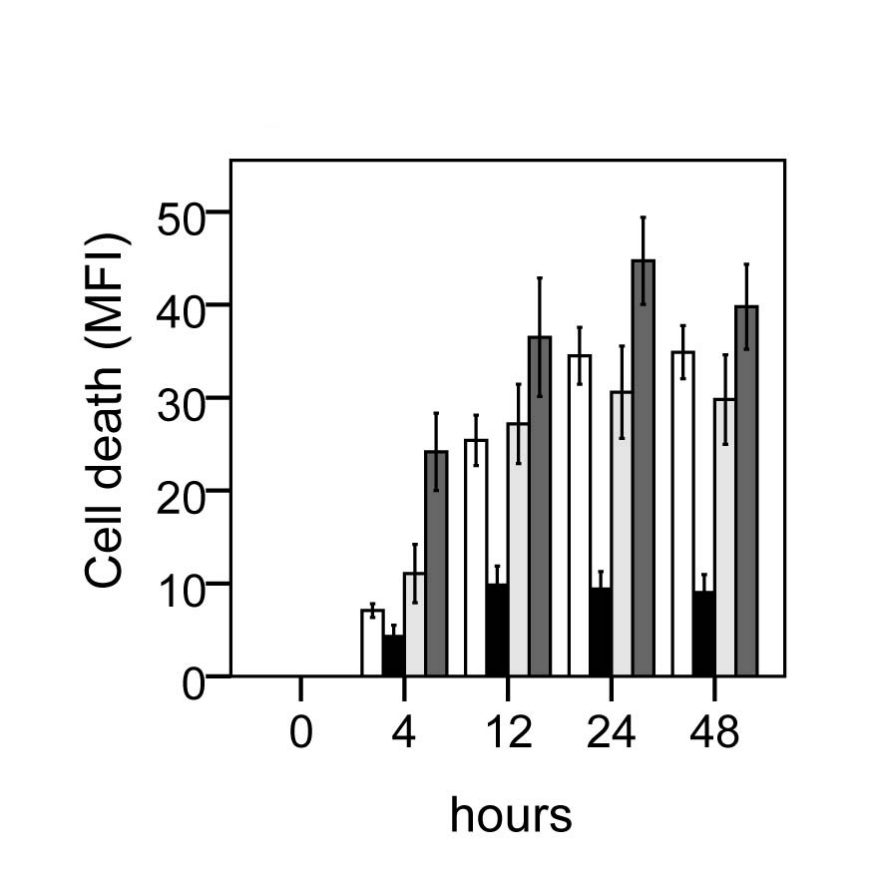
**Effect of β1-specific antagonist CGP 20712A or the specific-β2 antagonist ICI 118551 on the protective effect of dobutamine on neuronal cell death following lipopolysaccharide-induced sensitization to OGD in murine hippocampal slice cultures**. Cell death (MFI) after 24 h LPS + 15 min OGD (white bars), dobutamine (5 μM) +24 h LPS +15 min OGD (black bars), dobutamine (5 μM) + CGP 20712A (5 μM) + 24 h LPS + 15 min OGD (light shaded bars), dobutamine (5 μM) + ICI 118551 (5 μM) + 24 h LPS + 15 min OGD (dark shaded bars), at repeated points of recovery. Data are shown as means ± SEM with n = 18. Dobutamine (5 μM) + LPS + OGD *versus *LPS + OGD, p < 0.01 in the CA1; dobutamine (5 μM) + CGP 20712A (5 μM) + LPS + OGD *versus *dobutamine (5 μM) + LPS + OGD, p < 0.01 in the CA1; dobutamine (5 μM) + ICI 118551 (5 μM) + LPS + OGD *versus *dobutamine (5 μM) + LPS + OGD, p < 0.01 in the CA1 Repeated-measures ANOVA with *post hoc *Bonferroni.

Effect of adrenergic agonists on neuronal cell death following LPS+OGD was evaluated by co-incubating slices with the respective adrenergic agonists during the 24 h of LPS exposure, Figure 2. The β1-agonist dobutamine and the β2-agonist terbutaline both conferred protection to neuronal damage following LPS+OGD, Figure 2 and 3. At a concentration of 5 μM, dobutamine offered a 65% protection (p < 0.01 respectively), which further improved (85%) at 50 μM (p < 0.01), Figure 2. This protection was completely obliterated by co-incubation with both the specific β-antagonist CGP 20712A at 5 μM (p < 0.001) and with the specific β2-antagonist ICI 118551 at 5 μM, Figure 4.

To investigate the effect of preferential β2-AR activation, slices were preincubated with 5 μM terbutaline. At this concentration terbulatine offered a 24% protection (p < 0.01 respectively), that was further increased to 61% at 50 μM (p < 0.01), Figure 3. The neuro-protective effect of dobutamine and terbutaline at equimolar concentrations was compared in a separate experiment. At both 5 and 50 μM the effect of dobutamine was more pronounced (2.7 fold at a concentration of 5 μM and 1.5 fold at a concentration of 50 μM) than that of terbutaline at corresponding concentrations (p < 0.01 respectively).

**Figure 5:**
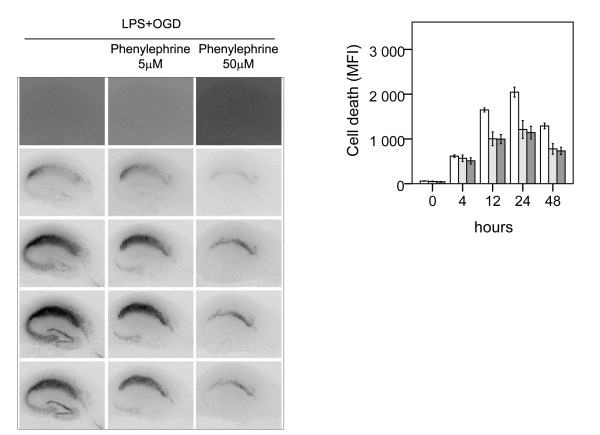
**Effect of α1-agonist phenylephrine on neuronal cell death following lipopolysaccharide-induced sensitization to OGD in murine hippocampal slice cultures**. A. Temporal development of cell damage (mean fluorescence intensity (MFI)) after 24 h LPS + 15 min OGD, phenylephrine (5 μM) +24 h LPS +15 min OGD and phenylephrine (50 μM) +24 h LPS + 15 min OGD. The same culture is shown at repeated time points of recovery. B. Cell death (MFI after 24 h LPS + 15 min OGD (white bars), phenylephrine (5 μM) +24 h LPS +15 min OGD (shaded bars), phenylephrine (50 μM) +24 h LPS + 15 min OGD (dark shaded bars), at repeated points of recovery. Data are shown as means ± SEM with n = 18. Phenylephrine (5 and 50 μM) + LPS + OGD versus LPS + OGD, p < 0.05 respectively in the CA1. Repeated-measures ANOVA with *post hoc *Bonferroni.

**Figure 6:**
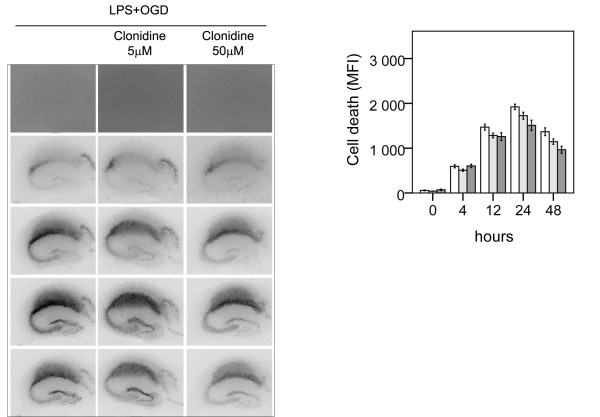
**Effect of α2-agonist clonidine on neuronal cell death following lipopolysaccharide-induced sensitization to OGD in murine hippocampal slice cultures**. A. Temporal development of cell damage (mean fluorescence intensity (MFI)) after 24 h LPS + 15 min OGD, clonidine (5 μM) +24 h LPS +15 min OGD and clonidine (50 μM) +24 h LPS + 15 min OGD. The same culture is shown at repeated time points of recovery. B. Cell death (MFI) after 24 h LPS + 15 min OGD (white bars), clonidine (5 μM) +24 h LPS +15 min OGD (shaded bars bars), clonidine (50 μM) +24 h LPS + 15 min OGD (dark shaded bars), at repeated points of recovery. Data are shown as means ± SEM with n = 18. Clonidine(5 and 50 μM) + LPS + OGD *versus *LPS + OGD, non-significant differences at both concentrations) in the CA1.

The α1-agonist phenylephrine offered a partial 44% protection (p < 0.05 respectively) with a similar effect at both concentrations (5 and 50 μM), Figure 5. In another set of experiments co-incubation of LPS with the α2-agonist clonidine (5 and 50 μM) did not affect neuronal cell death at either concentration, Figure 6.

### $\tilde{\beta }$-agonists depress cytokine levels in slices during exposure to LPS and following LPS and OGD

**Figure 7:**
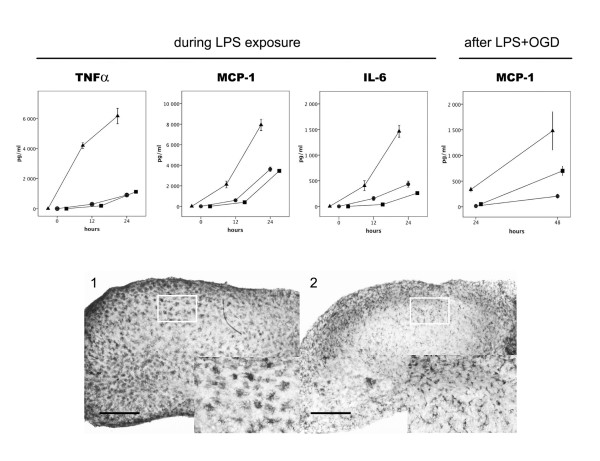
**β-adrenoceptor activation modulates lipopolysaccharide-induced inflammatory response before and after OGD in murine hippocampal slice cultures**. A. Mean concentrations of TNF-α, IL-6 and MCP-1 in culture medium at 12 and 24 h during 24 h of exposure to LPS (1 μg/ml) +/- co-incubation with β1-agonist dobutamine (50 μM) or β2-agonist terbutaline (50 μM): only LPS (triangle), LPS+ dobutamine (circle), LPS + terbutaline, (square). Far right, mean concentrations of MCP-1 at 24 and 48 h following 15 min OGD with pre-exposure to LPS for 24 h. N = 4 per experimental group at each time point. Error bars denote 1 SEM. Levels of TNF-α, IL-6 and MCP-1 were decreased by dobutamine and terbutaline (p < 0.01 respectively) during LPS exposure as where levels of MCP-1 following LPS+OGD (p < 0.01). Repeated-measures ANOVA with *post hoc *Bonferroni.B. F4/80 stained hippocampal slices illustrating microglial morphology. Immunohistochemistry was performed at 48 h after 15 min OGD with pre-exposure to LPS (1 μg/ml) for 24 h. (1) 24 h LPS + 15 min OGD, (2) 24 h LPS + β1-agonist dobutamine (50 μM) + 15 min OGD. Bars in 1 and 2 = 400 μm.

We have previously demonstrated that LPS exposure is associated with an increase in the secreted levels of TNF-α, IL-6 and MCP-1 at 12 and 24 h. Further, levels of MCP-1 were increased at 24 and 48 h after LPS exposure followed by OGD as compared with pure OGD [17]. The effects of co-incubation with dobutamine (50 uM) and terbutaline (50 uM) respectively on cytokine secretion during LPS exposure and following OGD were evaluated, Figure 7A. Secreted levels of the pro-inflammatory cytokines TNFα, MCP-1, and IL-6 in medium were decreased at 12 and 24 h during LPS exposure by dobutamine and terbutaline (p < 0.01 respectively) as where levels of MCP-1 at 24 and 48 h following LPS + OGD (p < 0.01 respectively). The suppressive effects of the respective β-agonists in equi-molar concentrations on cytokine secretion during LPS exposure and following LPS + OGD were similar.

### Dobutamin depresses LPS- and OGD-induced microglial activation

Hippocampal organotypic cultures from the respective groups were stained with the microglial marker F4/80 at 12 h following start of LPS exposure and at 24 and 48 h after OGD. Changes in staining intensity for F4/80 and morphology have been described as early sensitive indicators of microglial activation [21]. Control cultures displayed characteristically quiescent microglia, with ramified cell bodies at all time points. LPS-exposed cultures exhibited microglia with a swollen ameboid morphology at 24 h after OGD which was more pronounced at 48 h with microglia presenting a rough, densely stained plasma membrane. Pure OGD resulted in a characteristic distribution of activated microglia that was localized to the CA1-CA3 cell band. Incubation with dobutamine during the 24 h of LPS incubation resulted in the induction of a microglial morphology, which is unlike that observed in both activated and resting microglia characterised by ramified microglia with swollen cell bodies. Representative illustrations of immuno-staining with F4/80 in hippocampal slices are given in Figure 7B.

### Effect of β1-agonist on pure OGD and in hippocampal slices devoid of TNFR1 and TNFR2

**Figure 8:**
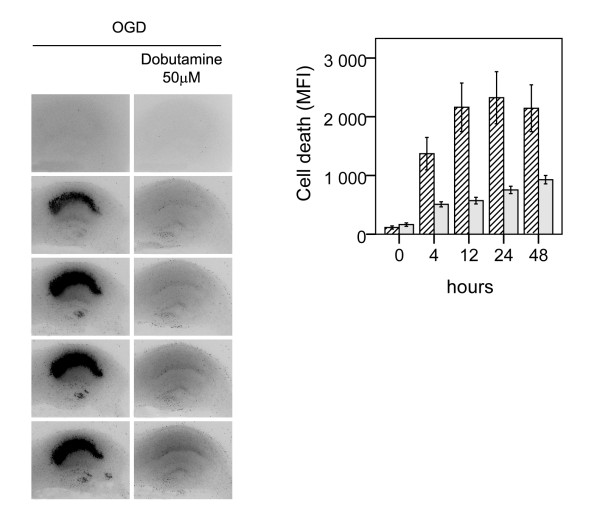
**Effect of β1-agonist dobutamine on neuronal cell death following OGD in murine hippocampal slice cultures**. A. Temporal development of cell damage (mean fluorescence intensity (MFI)) after 15 min oxygen-glucose deprivation (OGD) and β1-agonist dobutamine (50 μM) + OGD. The same culture is shown at repeated time points of recovery. B. Cell death (MFI) after 15 min OGD (hatched bars) and dobutamine (50 μM) + 15 min OGD (shaded bars) at repeated points of recovery. Data are shown as means ± SEM with n = 18. Dobutamine (50 μM) + OGD *versus *OGD, p < 0.01 in the CA1. Repeated-measures ANOVA with *post hoc *Bonferroni.

**Figure 9:**
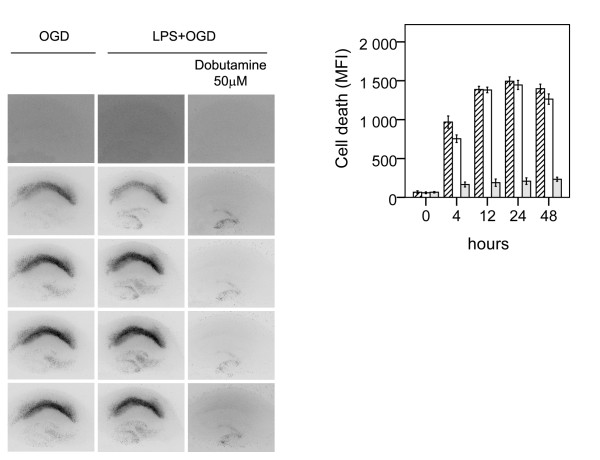
**Effect of β1-agonist dobutamine on neuronal cell death following lipopolysaccharide-induced sensitization to OGD in murine hippocampal slice cultures from TNFR1^-/- ^mice**. A. Temporal development of cell damage (mean fluorescence intensity (MFI)) after 15 min oxygen-glucose deprivation (OGD), LPS (1 μg/ml) for 24 h + 15 min oxygen-glucose deprivation (OGD) and β1-agonist dobutamine (50 μM) + 24 h LPS + OGD in murine hippocampal slice cultures from TNFR1^-/-^mice. The same culture is shown at repeated time points of recovery. B. Cell death (MFI) after 15 min OGD (hatched bars), 24 h LPS+ 15 min OGD (white bars) and dobutamine (50 μM) + 24 h LPS + 15 min OGD (shaded bars) at repeated points of recovery. Data are shown as means ± SEM with n = 18. Dobutamine (50 μM) + LPS + OGD *versus *LPS + OGD, p < 0.01 in the CA1. Repeated-measures ANOVA with *post hoc *Bonferroni.

**Figure 10:**
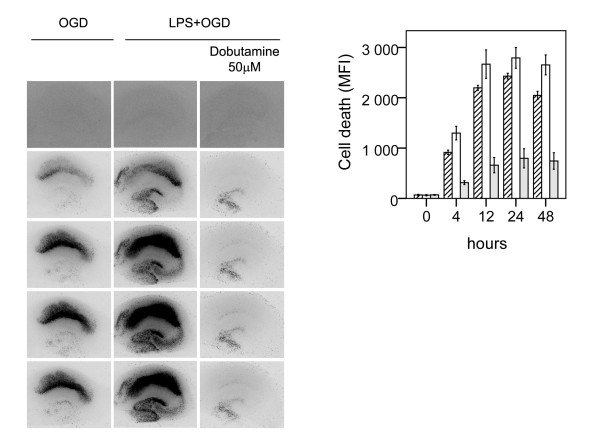
**Effect of β1-agonist dobutamine on neuronal cell death following lipopolysaccharide-induced sensitization to OGD in murine hippocampal slice cultures from TNFR2^-/- ^mice**. A. Temporal development of cell damage (mean fluorescence intensity (MFI)) after 15 min oxygen-glucose deprivation (OGD), LPS (1 μg/ml) for 24 h + 15 min oxygen-glucose deprivation (OGD) and β1-agonist dobutamine (50 μM) + 24 h LPS + OGD in murine hippocampal slice cultures from TNFR2^-/-^mice. The same culture is shown at repeated time points of recovery. B. Cell death (MFI) after 15 min OGD (hatched bars), 24 h LPS+ 15 min OGD (white bars) and dobutamine (50 μM) + 24 h LPS + 15 min OGD (shaded bars) at repeated points of recovery. Data are shown as means ± SEM with n = 18. Dobutamine (50 μM) + LPS + OGD *versus *LPS + OGD, p < 0.01 in the CA1. Repeated-measures ANOVA with *post hoc *Bonferroni.

Pre-incubation of slices with dobutamine (50 μM) 24 h prior to pure OGD without pre-exposure to LPS, resulted in a near total protection, 68%, without any visible cell death (p < 0.001), Figure 8. As previously shown, LPS-exposure prior to OGD in TNFR1^-/- ^slices did not result in aggravated neuronal damage as compared to exposure to pure OGD whereas the sensitizing effect of LPS to OGD was present in TNFR2-/- slices [17]. Pre-incubation with dobutamine (50 μM) during LPS exposure prior to OGD in hippocampal slices from both TNFR1^-/- ^and TNFR2^-/- ^mice respectively resulted in reduced cell death by 86% and 71% respectively (all p < 0.01), Figure 9 and 10, respectively.

## Discussion

Together these results show that β-AR activation provides neuro-protection in the setting of LPS-induced inflammation and OGD in the immature murine hippocampus. The neuro-protective effect was associated with decreased levels of pro-inflammatory cytokines during LPS exposure and a change in the morphological appearance of microglia. In addition, β-AR activation exhibited neuro-protective properties in pure OGD without preceding inflammatory exposure and in hippocampal slices devoid of TNFR1.

Infection with pro-inflammatory exposure and hypoxia-ischemia are both recognized as factors causing white and grey matter brain damage during the transition from fetal to neonatal life. The aggravating effect of induced inflammation on hypoxic-ischemic neuronal damage has been well documented *in vivo *[15]. We have previously characterized the sensitizing effect of LPS exposure on neuronal damage following OGD in juvenile murine hippocampal slice cultures and showed that time separation between the respective insults was decisive for resulting neuronal damage, *ie *for a sensitizing, or with an increased time separation, a protective effect [17]. Thus, this *in vitro *organotypic hippocampal slice culture system resembles *in vivo *experimental models in an essential aspect and is suitable for mechanistic studies.

### Neuro-protective effect is β1- and β2-adrenoceptor specific

Co-incubation of hippocampal slices during LPS exposure with the β1-agonist dobutamine and the β2-agonist terbutaline respectively resulted in a dose-dependent reduction of neuronal cell death following OGD. However, at equi-molar concentrations the effect of dobutamine was more pronounced than that of terbutaline with dobutamine offering almost complete protection at the higher concentration. However, co-incubation of dobutamine with the β1 antagonist CGP 20712A or with the β2 antagonist ICI 118551 clearly reversed the protective effect. Therefore, it seems that the observed protective effect is mediated via the β1- and the β2 receptors. As both receptors activate adenylate cylcase, the likely mechanism is an increase in intracellular cAMP levels that follow activation of either receptor.

Our results are in agreement with previous study showing that both β1 and β2 agonists may produce neuroprotection against glutamate induced excitotoxicity in mixed neuronal-glial cultures *in vitro *[22]. However, an *in vivo *study by the same group, provided evidence that the β2 AR is primarily responsible for the protective effect provided by adrenergic stimulation in a model of permanent middle cerebral artery occlusion in adult mice [22]. β1 receptors are found at higher densities than β2 receptors in normal mouse hippocampus [23], however, β2 ARs are upregulated on astrocytes following ischemia [24] which is accompanied by astrocyte activation. *In vitro *studies show that both β1 and β2 ARs may induce astrocyte activation [22]. It seems both β1 and β2 ARs are involved in neuroprotection following ischemia and both astrocytes and neurons, as well as immune cells are targeted by adrenergic stimulation [25].

The α1-agonist phenylephrine offered protection following induced inflammation and ischemia, however at a magnitude lower than that found with either β-agonist. The α2-agonist clonidine did not exhibit any discernible neuro-protective effects.

### Immunomodulatory effects of β-adrenoceptor stimulation

Co-incubation with both $\tilde{\beta }$agonists, terbutaline and dobutamine, during LPS-exposure caused a profound down-regulation of secretion of TNF-α, IL-6 and MCP-1 and morphological signs of modified microglial activation. The immuno-suppressive effect of β-AR stimulation has been extensively studied in several brain cell populations [3,4,26]. Binding to $\tilde{\beta }$AR and activation of c-AMP dependent pathways has been shown to inhibit the expression of numerous pro-inflammatory cytokines [27], including TNFα in microglia [28,29]. The capacity of microglia and monocytes to produce TNF-α and IL-1β in response to LPS is efficiently reduced by β-AR stimulation as well as by other c-AMP elevating agents [3,4].

### Neuroprotective mechanisms induced by β-adrenoceptor stimulation

We have previously shown that signalling by TNF-α through TNFR1 is essential for LPS-induced sensitization or pre-conditioning to OGD and resulting severely aggravated or reduced neuronal cell death. This suggests that the immune modifying effect of β-agonist exposure and, more specifically, the down-regulation of TNF-α secretion, was critical for the observed neuro-protective effect in the applied model of LPS-induced sensitization to OGD. Although this was not tested, β-AR activation may have been associated with increased neuronal cell death if evaluated in the pre-conditioning model applying a prolonged time-interval between insults.

The neuro-protective effect of β-AR activation in both pure OGD and in the combined insult LPS+OGD in slices devoid of TNFR1 suggests that β-AR activation influences detrimental mechanisms other than down-regulation of TNF-α. Recent studies have shown that β-AR activation increases synthesis of the anti-oxidant glutathione in cultured neurons as well as in mesenchymal stem cells rendering protection against oxidative stress [30,31]. Reinforcement of endogenous anti-oxidant production may be a mechanism whereby β-AR activation offered neuroprotection against both LPS-induced inflammation and OGD in our hippocampal slice culture model.

The release of glutamate during OGD and the resulting activation of excito-toxic mechanisms causing neuronal cell death are well documented in this hippocampal slice culture system [17]. We have previously shown that co-incubation with the NMDA-receptor antagonist MK-801 efficiently protected all studied neuronal subregions from cell death after pure OGD as well as that occurring after OGD with LPS pre-exposure. Hypothetically, a direct interference with NMDA-receptor activation by β-AR activation could explain the profound neuroprotective effects observed in the present study. Selective lesion of locus coeruleus, with a subsequent decrease in hippocampal adrenergic input, greatly augments neuronal cell death in the CA1 region following global cerebral ischemia in the adult rat [32]. In addition, β-AR activation has been shown to accelerate hypoxic inhibition of synaptic transmission in the hippocampal CA1 region through an increase in synaptic levels of adenosine thereby inhibiting glutamate release by activating adenosine A1 receptors [33]. In summary, the observed neuro-protective effect of β-AR activation may include several mechanisms of excitatory inhibition during OGD.

The individual capacity to increase levels of β-adrenergic agonists within the brain during exposure to inflammation and ischemia may be decisive for resulting degree of brain damage and functional impairment. Thus activation of β-ARs by increased levels of NE may be essential for endogenous neuro-protection. Extracellular levels of NE in the hippocampus increase 30-fold during global ischemia in the adult rat brain [34]. A corresponding increase of NE during ischemia in the immature brain has to our knowledge not been studied. However, the huge surge of circulating NE and E released by the adrenomedullary system during fetal hypoxia suggests a corresponding increase within the immature brain during ischemia.

The organotypic hippocampal slice culture model incorporates different cellular populations involved in the coordinated response to pro-inflammation and ischemia *within *the brain. Thus the present results were not biased by perturbations in the cardio-vascular system. However, brain ischemia and systemic inflammation *in vivo *comprise, apart from hemodynamic adaptations, a primary induction of mechanisms at the blood - endothelium interface with messengers evoking a secondary intra-cerebral response. Continued study will evaluate the possible neuro-protective effects of β-adrenergic activation taking in to consideration the interaction with the blood-brain barrier *in vivo*.

## Conclusions

Modulation of CA levels during inflammation and ischemia may be a powerful means to achieve neuro-protection. Continued study of mechanisms whereby CA mediate their neuro-protective effects may provide information useful for development of neuro-protective strategies.

## List of abbreviations used

AR: Adrenoceptor; CA1: Cornu ammonis 1; CA: Catecholamine; cAMP: Cyclic adenosine monophosphate; DG: Dentate gyrus; HBSS: Hank's balanced salt solution; IL-6: Interleukin-6; LPS: Lipopolysaccharide; MCP-1: Monocyte chemoattractant protein 1; MEM: Modified Eagle's medium; MFI: Mean fluorescence intensity; NE: Norepinephrine; OGD: Oxygen-glucose deprivation; TNF: Tumor necrosis factor; TNFR: Tumor necrosis factor receptor; PBS: Phosphate-buffered saline; PI: Propidium iodide

## Declaration of competing interests

The authors declare that they have no competing interests.

## Authors' contributions

TM contributed to design of the study, acquisition of data, analysis and interpretation of data and drafting the manuscript. SRH contributed to the design of the study, interpretation of data and drafting of the manuscript. TC contributed to the design of the study, interpretation of data and drafting of the manuscript. CC contributed to the design of the study, interpretation of data and drafting of the manuscript. TW contributed to design of the study, analysis and interpretation of data and drafting the manuscript. DL contributed to design of the study, acquisition of data, analysis and interpretation of data and drafting the manuscript. All authors have read and approved the final manuscript.
